# The diagnostic and predictive potential of lncRNA CASC2 targeting miR-155 in systemic lupus erythematosus patients with nephritis complication

**DOI:** 10.1038/s41598-024-81212-5

**Published:** 2024-12-17

**Authors:** Nada R. Mohamed, Abeer l. Abd El-Fattah, Olfat Shaker, Ghadir A Sayed

**Affiliations:** 1 Department of Biochemistry, Faculty of Pharmacy, Egyptian Russian University, Cairo, Egypt; 2 Department of Biochemistry and Molecular Biology, Faculty of Pharmacy (Girls), Al-Azhar University, Cairo, Egypt; 3 Department of Medical Biochemistry and Molecular Biology, Faculty of Medicine, Cairo University, Cairo, Egypt

**Keywords:** Lupus nephritis, MiR-155, LncRNA CASC2, SLE diagnosis, LN prediction, Biochemistry, Genetics

## Abstract

Lupus nephritis (LN) is a serious problem that results from systemic lupus erythematosus (SLE) complications. Recent studies have highlighted that non-coding RNA (ncRNA) dysregulation is a notable feature in patients with SLE. As a result, this research was designed to investigate lncRNA CASC2 and miR-155 levels as non-invasive diagnostic biomarkers in SLE patients, including those with and without nephritis, and to investigate their effectiveness in assessing disease severity and predicting LN. Our study included 60 patients with SLE who were subclassified into (30 non-LN and 30 LN groups), along with 30 control subjects. Quantification of lncRNA CASC2 and miR-155 in serum samples from the Egyptian population was carried out with real-time polymerase chain reaction (RT-PCR). The disease activity index (SLEDAI) for SLE was evaluated, and the analysis of the receiver operating characteristic (ROC) curve was implemented. Increased levels of lncRNA CASC2 were observed in SLE patients compared to healthy controls, with even higher levels observed in the LN group versus the non-LN patients’ group. Conversely, miR-155 was noted to be down-regulated in SLE patients relative to controls, and its levels were lower in the LN group relative to the non-LN patients’ group. The elevated expression of lncRNA CASC2 and reduced expression of miR-155 were both correlated to the severity of the disease. The current study illustrated that both lncRNA CASC2 and miR-155 could act as valuable non-invasive diagnostic biomarkers for SLE and predicting LN among SLE patients, as well as their abilities to detect the disease severity and progression.

## Introduction

Systemic lupus erythematosus (SLE) is an autoimmune condition marked by the presence of antibodies targeting nuclear and cytoplasmic antigens, resulting in widespread systemic impairment^1^. Numerous organs, including the hematopoietic system, nervous system, joints, skin, heart, and kidneys are impacted by SLE disease^2^. Lupus nephritis (LN) is the most prevalent organ-specific complication of this autoimmune condition, affecting approximately 50–60% of patients with lupus^3^. It is a complicated process that results in autoantibodies deposition in the glomerulus, complements and macrophages activation, production of proinflammatory cytokines and chemokines, and cell proliferation, which cause tubulointerstitial inflammation and tubular damage^4^.

The guidelines recommend renal biopsy as the most reliable method for LN diagnosing, as it provides definitive histopathological confirmation^5^. However, this procedure is invasive, carries risks such as bleeding, and is difficult to repeat^6^. Currently, commonly utilized laboratory markers for diagnosing SLE, particularly LN include, antinuclear antibodies (ANA), anti-double-stranded deoxyribonucleic acid (anti-dsDNA) antibodies, serum complements (C3 and C4), serum creatinine (SCr), urinary protein, and glomerular filtration rate^7,8^. However, owing to their inadequate sensitivity and specificity, these clinical parameters are unable to satisfy the practical requirements of clinical settings^7^. Thus, it is critical to discover innovative non-invasive biomarkers that can diagnose SLE, identify LN activity, predict renal flares earlier, and estimate the disease progression^9^.

Current studies have shown the outstanding roles of non-coding ribonucleic acids (ncRNAs) in SLE pathogenesis^10,11^. Regulatory ncRNAs are categorized into two main types based on their length: small ncRNAs, which are shorter than 200 nucleotides (nt) and include microRNAs (miRNAs), and long ncRNAs (lncRNAs), which are longer than 200 nt^12^.

Long noncoding RNA cancer susceptibility candidate gene 2 (CASC2) is an oncogenic lncRNA positioned on chromosome 10 of the human genome and has been implicated in several types of cancer, as it plays a fundamental role in cancer biology by influencing the cell behaviors, including proliferation, invasion, and migration^13^.

Previous studies have pointed to the dysregulation of lncRNA CASC2 in various diseases associated with immunity dysregulation, inflammation, and organ damage^14–16^. For example, increased levels of lncRNA CASC2 have been observed in osteoarthritis patients, suggesting that CASC2 is a key regulator of Interleukin-17 (IL-17) expression and chondrocyte proliferation and apoptosis^17^. It was also reported that high expression of CASC2 was positively correlated with astrocytoma progression, considering it as a poor prognostic marker for tumorigenic patients^18^. In addition to its role in the inflammation and apoptosis of fibroblast-like synoviocytes of rheumatoid arthritis patients^19^.

Notably, lncRNACASC2 has been shown to target essential molecules involved in regulating inflammatory or immune responses. It is involved in modulating different inflammatory signaling pathways that are central to SLE pathology, including nuclear factor kappa B (NF-κB), suppressor of cytokine signaling 2 (SOCS2), and mitogen-activated protein kinase (MAPK) signaling pathways^20–22^, and control the expression of proinflammatory cytokines such as IL-17, IL-6, IL-18 and tumor necrosis factor-alpha (TNF-α)^23,24^.

Furthermore, subsequent studies have discussed the potential connection between CASC2 and kidney involvement which is the main complication of SLE such as diabetic nephropathy and sepsis‑induced acute kidney injury^22,25^. However, to the best of our knowledge, no previous reports have established a direct link between lncRNA CASC2 and SLE specifically LN, and therefore, the current study has revealed lncRNA CASC2 as a promising candidate for further exploration.

MicroRNA-155 is a typical multifunctional miRNA transcribed from the B cell integration cluster located on chromosome 21 and involved in hematopoiesis, inflammation, and immune responses^26^. MiR-155 has been identified as both a pro-inflammatory and anti-inflammatory mediator that influences immune response modulation^27^.

Regarding its role as an anti-inflammatory mediator, *Yang et al.*^28^ hypothesized that the elevation in miR-155 level might suppress the NF-κB signaling cascade, which in turn mitigates inflammation, oxidative stress, and apoptosis triggered by IL-1β in rat nucleus pulposus cells. Another study revealed the downregulation of miR-155 in neutrophils and a tendency toward decreasing in lymphocytes of patients with SLE^29^.

In contrast, *Chen et al.*^30^ observed that the miR-155 level was significantly elevated in peripheral blood mononuclear cells (PBMCs) of patients diagnosed with SLE compared to those of healthy controls. Increased miR-155 levels have also been observed in SLE patients’ macrophages, dendritic cells, B cells, and T cells^31^. Furthermore, it helps to produce inflammatory T cells, such as T helper 17 (Th17) and Th1 subsets that promote tissue inflammation^32^.

Recently, the competitive endogenous RNA (ceRNA) regulatory hypothesis described lncRNA as miRNA sponges that compete with an mRNA for binding to the same miRNA, thus reducing their regulatory effect on mRNAs^33^. Several studies confirmed that CASC2 can directly bind to miR-155 to inhibit its expression in various pathological processes^25,34^. For instance, *He et al.*^35^. found that lncRNACASC2 overexpression could promote fibroblast proliferation and migration, and inhibit apoptosis via targeting miR-155-5p, thus facilitating wound healing in diabetic foot ulcers. In addition, lncRNACASC2 could act as a ceRNA for miR-155, regulating the signal transducer and activator of transcription 3 (STAT3) expression and inhibiting the proliferative and metastatic capability of colorectal cancer cells^36^.

Consequently, this study was designed to explore the serum levels of lncRNA CASC2 as a novel diagnostic biomarker in SLE and its correlation with its downstream target miR-155 expression levels in SLE patients (with and without nephritis), explore the correlation of both biomarkers with the clinicopathological parameters of SLE, also to determine their ability to detect disease severity and progression and predict LN among SLE patients.

## Subjects and methods

### Clinical data collection

The study comprised sixty SLE patients aged between 18 and 60 years, all of whom met the diagnostic criteria for SLE established by the Systemic Lupus International Collaborating Clinics (SLICC)^37^. Thirty patients (non-LN group) had shown no signs of renal involvement since the onset of the disease, as evidenced by proteinuria < 500 mg/day and the absence of urinary sediment, including hematuria and/or cellular casts, while the other thirty patients (LN group) exhibited impaired kidney function, with proteinuria > 500 mg/day, presence of active urine sediment, and/or an increase in SCr level. Renal involvement was confirmed through renal biopsy. The control group involved thirty apparently healthy volunteers matched in age and sex. We excluded patients diagnosed with any other autoimmune inflammatory disorders, malignancy, chronic infections, chronic diseases, or a history of nephrotoxic drugs from this study. The patients were registered in the rheumatology outpatient clinic located at Kasr EL-Aini Hospital, from November 2023 to May 2024. All participants underwent a comprehensive history-taking and clinical examination, and their SLE Disease Activity Index (SLEDAI) score was assessed^38^. Renal biopsies were performed on all LN patients. Histopathology staging was classified according to the 2004 International Society of Nephrology/Renal Pathological Society (ISN/RPS) classification into class I (minimal mesangial LN), class II (mesangial proliferative LN), class III (focal LN), class IV (diffuse LN) and class V (membranous LN). This study was granted approval by the Medical Ethical Committee at the Faculty of Pharmacy (Girls), Al-Azhar University (REC: 430:8\2023) and the Medical Ethical Committee at the Faculty of Pharmacy, Cairo University (REC: BC (3566):3\2024). All patients signed informed consent that corresponded to the Declaration of Helsinki’s ethical principles before participating in the study.

**Bioinformatics**: The lncRNA CASC2 and miR-155 interaction had been predicted utilizing the ENCORI database (https://rnasysu.com/encori/). TargetScan (https://www.targetscan.org/vert_80/) was performed to determine the predicted target genes of miR-155.

### Serum samples collection

For RNA extraction, a venous blood sample was drawn from all participants and placed in a centrifuge tube for serum separation, then it was kept at -80 °C until further analysis.

### Routine laboratory tests

Routine laboratory tests were conducted, including the measurement of erythrocyte sedimentation rate (ESR) utilizing the Westergren technique^39^, and the assessment of serum C-reactive protein (CRP) levels via enzyme-linked immunosorbent assay (ELISA) technique utilizing (Accu-Bind™ ELISA Microplate Test System Monobind Inc., USA) (catalog no.3125 − 300), in compliance with *Kindmark*^40^. Immunological markers including, serum C3 and C4 that were measured through the complement C3 and C4 Assay Kits (Weldon Biotech India Pvt. Ltd.) (catalog no. GB670M, GB680M) respectively, ANA that was estimated using ANA-8-Screen ELISA kit (DRG Diagnostic GmbH, Marburg, Germany) (catalog no. EIA-3562), anti-dsDNA antibody was detected via anti-dsDNA IgG ELISA kit (DRG Diagnostic GmbH, Marburg, Germany) (catalog no. EIA-6137) and Lupus anticoagulant (LAC) was determined by human LAC ELISA Kit (Abbexa LTD, Cambridge, UK) (catalog no. abx052842). Renal specific-markers including, SCr that was determined spectrophotometrically via the NanoDrop ND-100 spectrophotometer (Thermo Fisher Scientific, Inc., Waltham, MA, USA), and Proteinuria levels were determined by the turbidimetric method employing the Biosystems BTS-350 spectrophotometer (Barcelona, Spain), following the manufacturer’s recommendations^41^.

### Quantification of lncRNA CASC2 and miR-155 expression levels using quantitative real-time polymerase chain (qRT-PCR) reaction

#### Serum RNA extraction

The extraction of total RNA from 5 ml serum samples was performed with QIAzol lysis reagent (Qiagen^®^, USA) and miRNeasy Mini Kits (217004; Qiagen^®^, Hilden, Germany), following the instructions provided by the manufacturer. RNA concentration and purity were then quantified with the NanoDrop^®^ spectrophotometer (Thermo Scientific^®^, USA).

### Reverse transcription reactions

For the synthesis of complementary DNA (cDNA), the extracted RNA was processed through reverse transcriptase (RT)-polymerase chain reaction (PCR) with the miScript II RT Kit (catalog no. 218161) from (Qiagen, Hilden, Germany), as directed by the manufacturer. The process involved polyadenylation of RNA with Poly (A) Polymerase followed by reverse transcription into cDNA, performed on an Applied Biosystems^®^ 2720 at 37 °C for 60 min, followed by 95 °C for 5 min (Bioline, Singapore, USA).

### Quantitative real-time polymerase chain reaction

The qRT-PCR amplification of cDNA templates was done using the miScript SYBR Green PCR Kit (Qiagen, Germany) (catalog no. 218073). For miR-155, each reaction had a total volume of 25 µL and utilized the specific miR-155 primer (catalog no. MS00033712). U6 snRNA served as the normalization control for the relative quantification of miR-155. Whereas, for lncRNA CASC2, each reaction had a total volume of 20 µL and utilized the specific lncRNA CASC2 primer (catalog no. NR_026939). Expression normalization and relative quantification of lncRNA CASC2 were achieved using GAPDH, using the primer sequences: forward primer 5’-CCCTTCCCCGATTTCAACTT-3’ and reverse primer 5’TGGCCCATGGTGATGAAATT-3’. The qRT-PCR protocol for both targets was set up with an initial 15-minute activation step at 95 °C, followed by 15 s of denaturation at 95 °C, and a final combined annealing and extension step at 60 °C for 60 s. The steps were cycled 40 times through the Rotor-Gene qRT-PCR system (Qiagen, USA). Relative RNA expression was quantified using the 2^−ΔΔCt^ method^42^.

### Statistical analysis

Statistical computer package version 24.0 (SPSS) was applied for analyzing the collected data statistically. GraphPad Prism 7 (Softhead, Inc.) and MedCalc Software Ltd version 22.023 were used for the figures plot. The data of this study was non-parametric and displayed as median (minimum-maximum) and interquartile range (IQR) for quantitative data. The Mann-Whitney U test (U) was used for pairwise group comparisons, while the Kruskal-Wallis (KW) test was applied for comparisons across three groups. Except for the section on the comparison between the histopathological Stages in the LN group, the data are represented as means ± standard deviation (SD). The Independent-samples t-test was used for pairwise group comparisons, while the One-Way ANOVA test was applied for comparisons across three groups. Numerical values and percentages were utilized to illustrate the qualitative data. The significance was evaluated using the Chi-square (χ2) test. Whenever more than 20% of expected values were less than 5, the Fisher Exact test (FEX) was applied instead. Spearman’s correlation was run to study the possible correlation between ncRNAs and the studied parameters among each group. ROC curve analyses were conducted determining AUC, the optimal specificity, and sensitivity for serum lncRNA CASC2 and miR-155 to investigate their potency as a diagnostic and predictive tool. *P* values below 0.05 were identified as statistically significant differences.

## Results

### Demographic, laboratory and clinical characteristics of the studied groups

The demographic, laboratory and clinical data for all of the cases in this study are represented in (Tables 1 and 2). In the LN group (*n* = 30), there were 24 females (80%) and 6 males (20%), with ages varying between 18 and 50 years (median of 30 years). In the non-LN group (*n* = 30), 20 (66.7%) were female and 10 (33.3%) were male, with ages from 21 to 53 years (median of 36 years). The control group was thirty healthy people with an age (median of 33 years) and sex (73.3% females and 26.7% males), age and sex were matched in all groups (Table 1).

Regarding laboratory data, the LN group exhibited significantly higher levels of CRP, SCr, proteinuria and SLEDAI when compared with the non-LN group (*p* < 0.001 for each). Compared to the control group, the C3 and C4 levels were lower significantly in both the LN and non-LN groups (*p* < 0.001) Nevertheless, no statistically significant variations were observed in the levels of ESR, anti-dsDNA, C3, C4, or LAC among the LN and non-LN groups (Table 1). Different clinical manifestations could be used to diagnose systemic lupus. The current results showed that, with the exception of arthritis (*p* < 0.05), no significant differences were found between the patient subgroups (LN and non-LN). However, there was significant variation between the patient subgroups and the control group in terms of many clinical manifestations, including oral ulcers, arthritis, rash, photosensitivity, leukopenia, lymphopenia, thrombocytopenia and neurological features (*p* value < 0.05) (Table 2).

**Table 1:** Demographic and laboratory data of SLE patients with and without LN and healthy controls.

variables	Groups			Test of significance	*P* value
	Control group (*n* = 30)	(Non-LN) group (*n* = 30)	(LN) group (*n* = 30)		
Sex, n (%)					
Male Female	8 (26.7) 22 (73.3)	10 (33.3) 20 (66.7)	6 (20) 24 (80)	X^2^ = 1.364	0.506
Age					
Median (MIN-MAX)	33 (18–49)	36 (21–53)	30 (18–50)	KW = 4.818	0.090
ESR (mm/hr)					
Median (MIN-MAX)	7.75 (2–19)	35 (15–78) ^a^	36 (8-130) ^a^	KW = 52.681	0.000*
CRP (mg/dl)					
Median (MIN-MAX)	0.3 (0.03–0.8)	2.45 (1-4.2) ^a^	3.45 (1.2-6) ^a, b^	KW = 62.290	0.000*
SCr (mg/dl)					
Median (MIN-MAX)	0.6 (0.5-1)	0.7 (0.5–1.1) ^a^	1.03 (0.5–1.9) ^a, b^	KW = 25.041	0.000*
Proteinuria(g/day)					
Median (MIN-MAX)	0.03 (0-0.1)	0.03 (0-0.08)	1 (0–7) ^a, b^	KW = 52.293	0.000*
C3(µg/ml)					
Median (MIN-MAX)	154.5 (94.0-180)	80 (30–130) ^a^	68 (20-143.5) ^a^	KW = 53.684	0.000*
C4(µg/ml)					
Median (MIN-MAX)	36 (23–50)	19.6 (6.4–79.5) ^a^	15.65 (5.1–43) ^a^	KW = 42.529	0.000*
SLEDAI score					
Median (MIN-MAX)	-----	1 (0–7)	4.5 (0–20) ^b^	U = 293.00	0.017*
Renal biopsy class, n (%)					
II III IV V	-----	-----	6 (20) 6 (20) 14 (46.67) 4 (13.33)	-----	-----
ANA, positive pattern, 1:80	< 1\80	80 (80–640)	240 (80-1280) ^b^	U = 173.00	0.059
Speckled + homogenous + rim		23: 19 + 4 + 0	22: 11 + 10 + 1	FEX = 5.507	0.021*
Anti-dsDNA, n (%)					
-ve +ve	30 (100) 0 (0)	7^a^ (23.3) 23 (76.7)	8^a^ (26.7) 22 (73.3)	FEX = 6.487	0.000*
LAC, n (%)					
-ve +ve	30 (100) 0 (0)	19^a^ (63.3) 11 (36.7)	13^a^ (43.3) 17 (56.7)	FEX = 24.79	0.000*

The data are represented as the median (min-max) or number (percentage). X^2^: Chi Square test; FEX: Fisher Exact test; KW: Kruskal Wallis test; U: Mann-Whitney U test. a: Significant difference relative to the controls. b: Significant difference relative to the non-LN patients’ group.

ESR: erythrocyte sedimentation rate; CRP: C-reactive protein; SCr: serum creatinine; ANA: anti-nuclear antibody; C3&C4: complements 3&4; Anti-dsDNA: anti-double stranded deoxyribonucleic acid; LAC: lupus anticoagulant; SLEDAI: systemic lupus erythematosus disease activity index.

**Table 2:** Clinical data of the studied groups.

Variables	Groups			*P* value
	Control group (*n* = 30)	Non-Nephritis group (*n* = 30)	Nephritis group (*n* = 30)	
Oral ulcers, n (%)				
-ve +ve	30 (100) 0 (0)	18^a^ (60) 12 (40)	16^a^ (53.3) 14 (46.7)	0.000*
Arthritis, n (%)				
-ve +ve	30 (100) 0 (0)	8^a^ (26.7) 22 (73.3)	16^a, b^ (53.3) 14 (46.7)	0.000*
Rash, n (%)				
-ve +ve	30 (100) 0 (0)	13^a^ (43.3) 17 (56.7)	15^a^ (50) 15 (50)	0.000*
Photosensitivity, n (%)				
-ve +ve	30 (100) 0 (0)	21^a^ (70) 9 (30)	18^a^ (60) 12 (40)	0.000*
Leukopenia, n (%)				
-ve +ve	30 (100) 0 (0)	13^a^ (43.3) 17 (56.7)	17^a^ (56.7) 13 (43.3)	0.000*
Lymphopenia, n (%)				
-ve +ve	30 (100) 0 (0)	20^a^ (66.7) 10 (33.3)	21^a^ (70) 9 (30)	0.000*
Thrombocytopenia, n (%)				
-ve +ve	30 (100) 0 (0)	18^a^ (60) 12 (40)	21^a^ (70) 9 (30)	0.000*
Neurological, n (%)				
-ve +ve	30 (100) 0 (0)	25^a^ (83.3) 5 (16.7)	22^a^ (73.3) 8 (26.7)	0.004*

a: Significant difference relative to controls. b: Significant difference relative to the non-LN patients’ group.

### Comparative analysis of serum lncRNA CASC2 and miR-155 expression among studied groups

**Fig. 1:**
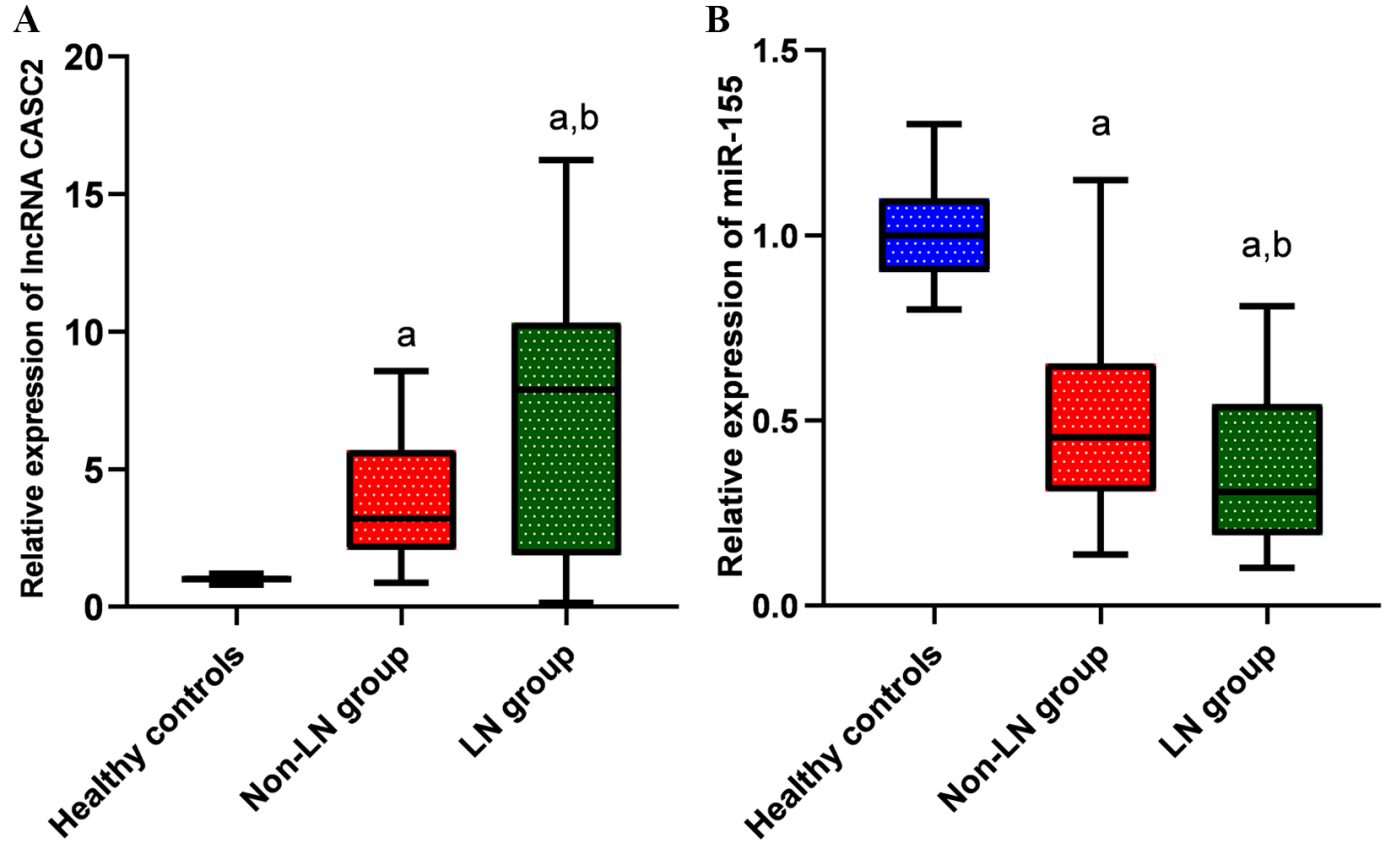
Biomarkers relative expression in the studied groups. (**A**) LncRNA CASC2 expression. (**B**) MicroRNA-155 expression. **a**: Significant difference relative to controls. b: Significant difference relative to the non-LN patient group. Statistical significance is considered at *P* < 0.05. Box and whisker plots: the box illustrates the interquartile range (25th to 75th percentiles), the line inside the box indicates the median, and the lower and upper lines denote the 10th and 90th percentiles of ncRNAs expression.

The serum level of lncRNA CASC2 was significantly increased in non-LN patients, showing a median (IQR) fold change of 3.21 (2.07–5.7) (*P* < 0.001) versus the healthy control group, and a higher value of 7.89 (1.87–10.33) (*p* < 0.01) was observed among the LN group relative to both the non-LN (*p* < 0.01) and control groups (*p* < 0.01). Conversely, miR-155 showed a significant decrease in the non-LN group, with a median (IQR) fold change of 0.45 (0.3–0.65) (*P* < 0.001) when compared to the control group, and an even lower value of 0.31 (0.19–0.54) (*P* < 0.01) was observed within the LN group versus both the non-LN (*p* < 0.05) and control groups (*p* < 0.01) as shown in (Fig. 1).

### Comparative analysis of lncRNA CASC2 and miR-155 expression in LN patients’ group across histopathological stages

**Fig. 2:**
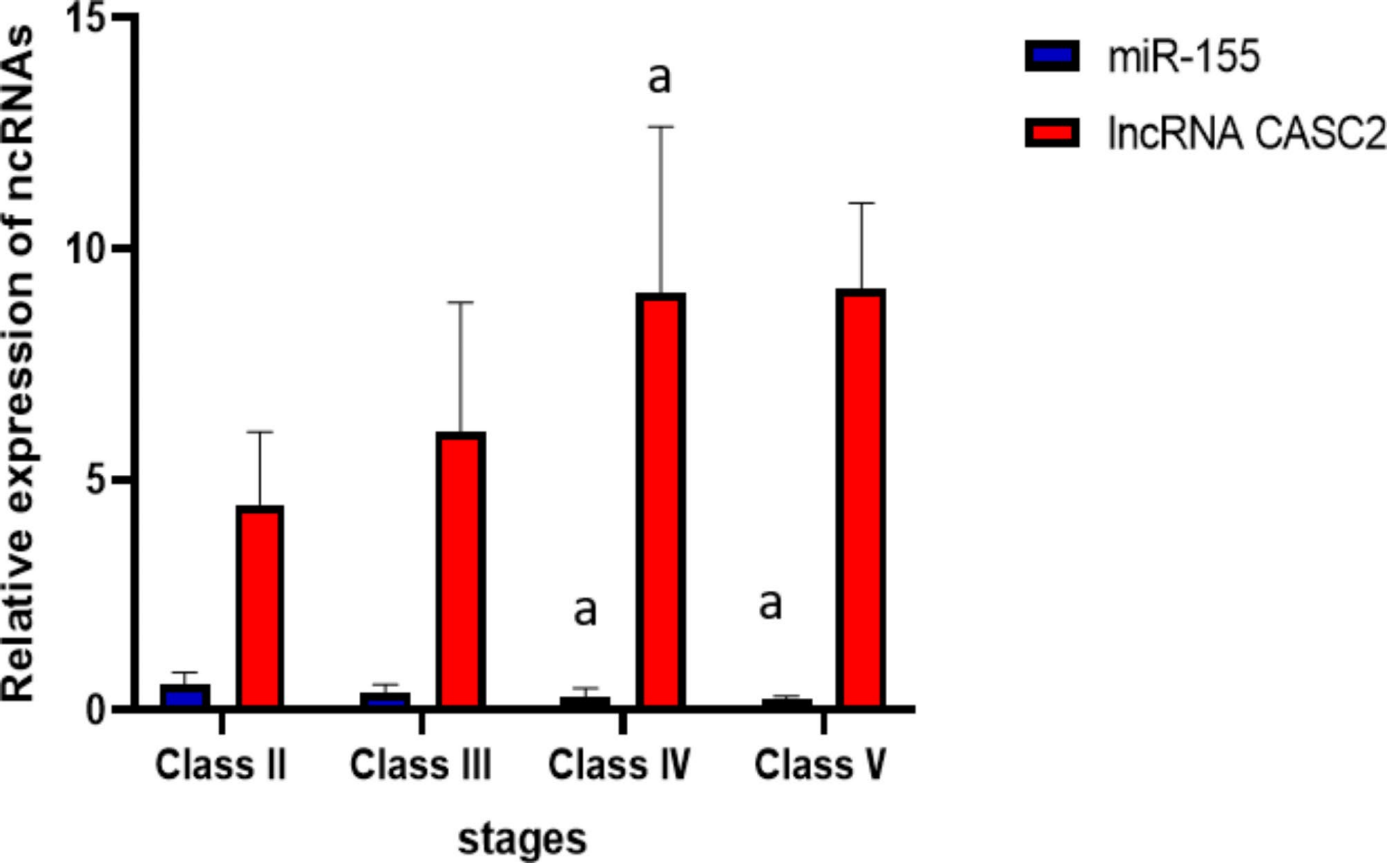
Biomarkers relative expression in the LN group at different histopathological stages.

When analyzing lncRNA CASC2 and miR-155 expression levels across different stages of LN, higher expression of lncRNA CASC2 was significantly correlated with the progression to more advanced stages of the disease (*p* < 0.05). Pairwise comparisons between stages revealed a significant difference between class II and IV (*p* < 0.05). Contrarily, the reduction in miR-155 expression level was significantly correlated with the later stages of the disease (*p* < 0.05). Pairwise comparisons between stages revealed a significant difference between class II and class (IV-V) (*p* < 0.05) as illustrated in (Fig. 2).

The data are represented as the mean ± SD. a: Significant difference relative to the class II group.

Statistical significance is considered at (*P* < 0.05).

### ROC curve analysis

**Fig. 3:**
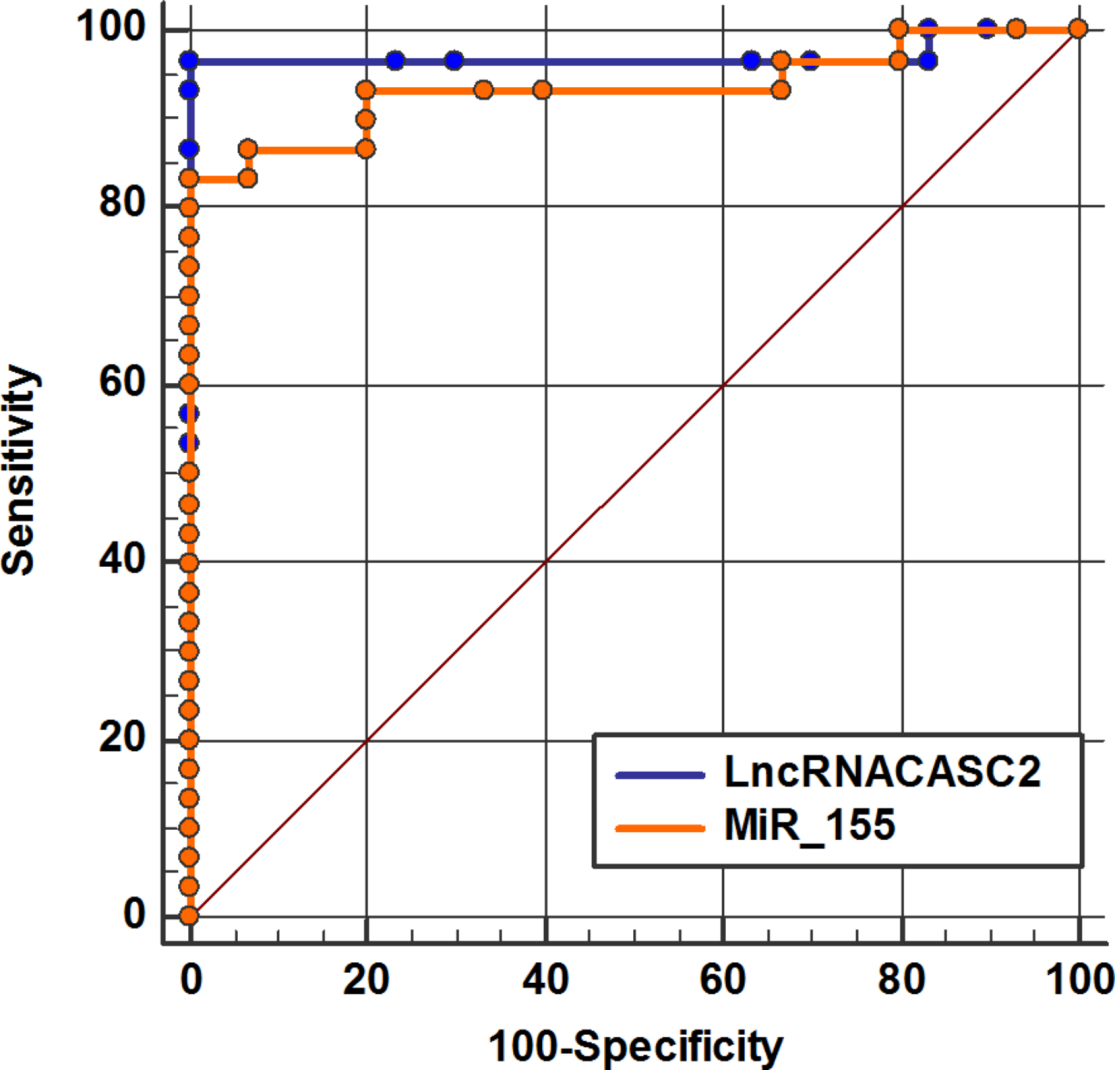
ROC curve analysis illustrates the diagnostic ability of lncRNA CASC2 and miR-155 in distinguishing the non-LN group from the control group.

**Fig. 4:**
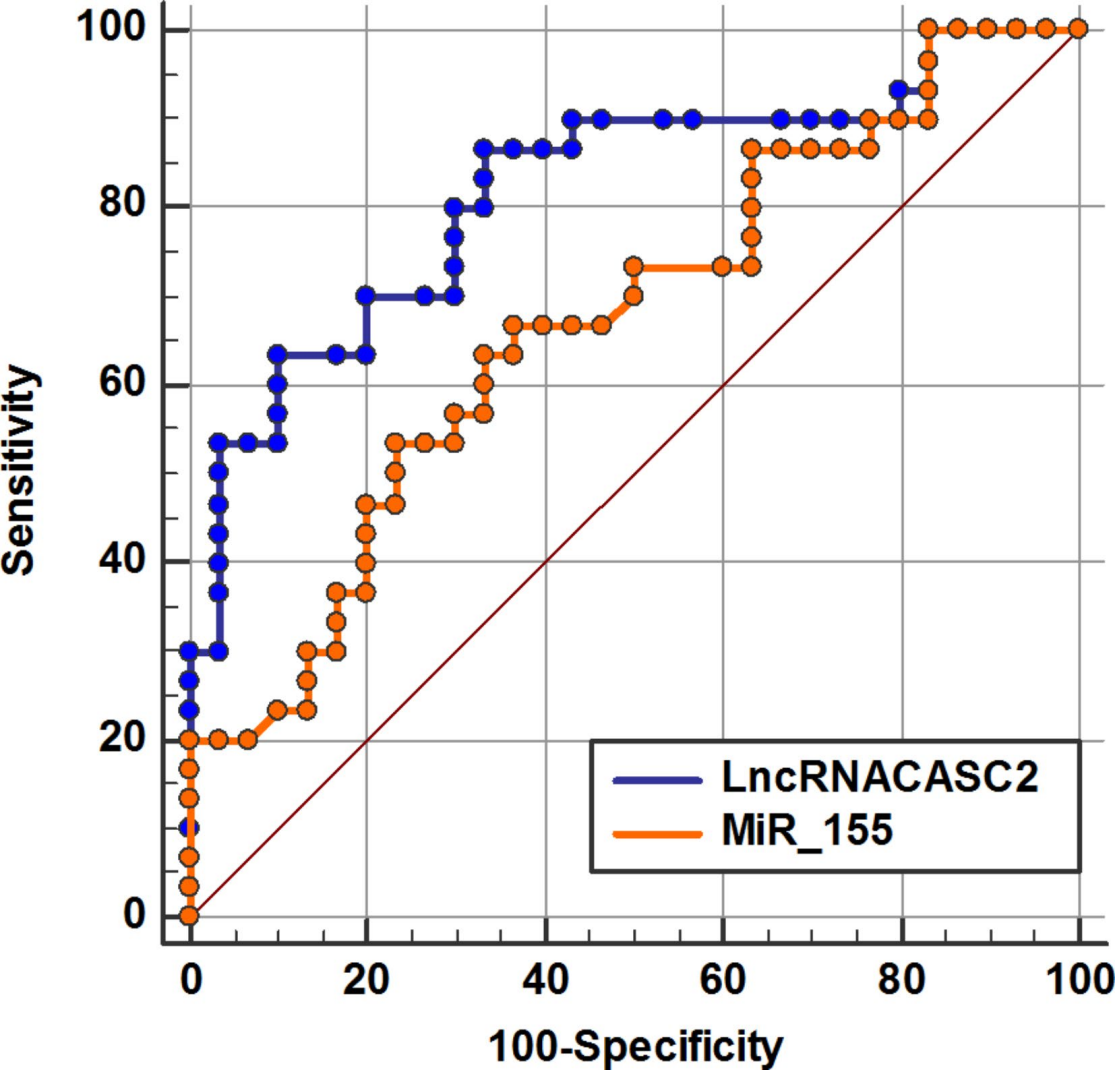
ROC curve analysis illustrates the predictive ability of lncRNA CASC2 and miR-155 in distinguishing non-LN from the LN group.

ROC analysis was carried out to explore if lncRNA CASC2 and miR-155 expression levels could differentiate between control and SLE patients’ groups. The AUC for lncCASC2 was 0.97, indicating 100% specificity and 96.67% sensitivity at the best cutoff value (1.24), while the AUC for miR-155 was 0.93, indicating 100% specificity and 83.33% sensitivity at the best cutoff value (0.76) as illustrated in (Fig. 3), These results indicated that both of them could be used as potential diagnostic biomarkers for SLE. ROC analysis was also conducted to distinguish between the LN and non-LN groups. Concerning lncRNA CASC2, the AUC was 0.82, indicating 66.7% specificity and 86.67% sensitivity at the optimal cutoff value (4.26), whereas for miR-155, the AUC was 0.67, indicating 63.3% specificity and 66.7% sensitivity at the optimal cutoff value (0.4) (Fig. 4). ROC curve indicated that lncRNA CASC2 had superior predictive activity for LN than miR-155.

### Linear regression analysis

**Fig. 5:**
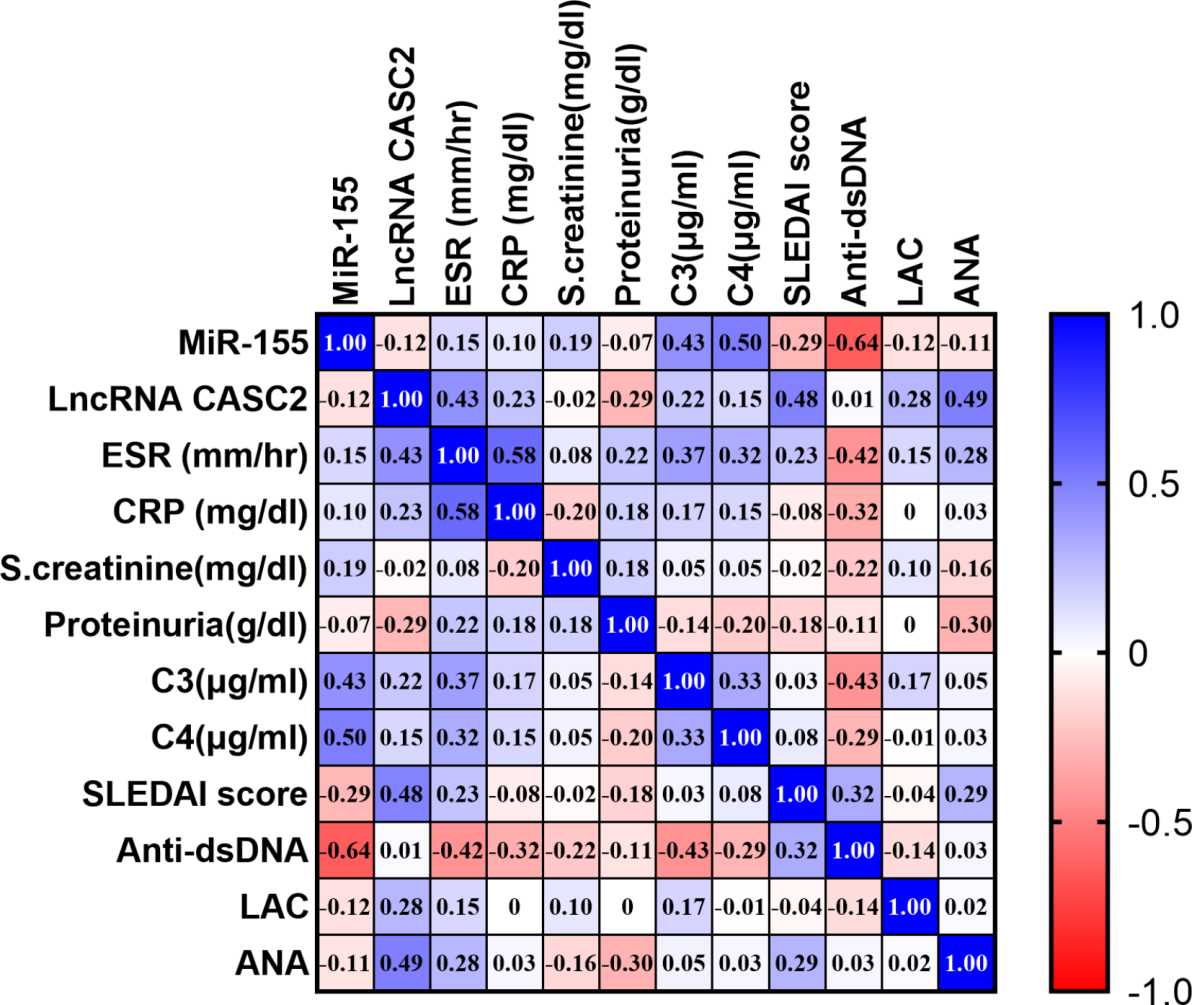
Spearman correlations among serum ncRNAs and laboratory variables in the non-LN group are illustrated on a correlation map. The map features a blue-red scale, with blue representing correlations near 1, red representing correlations near − 1, and white representing correlations around 0. Statistical significance at *P* < 0.05.

**Fig. 6:**
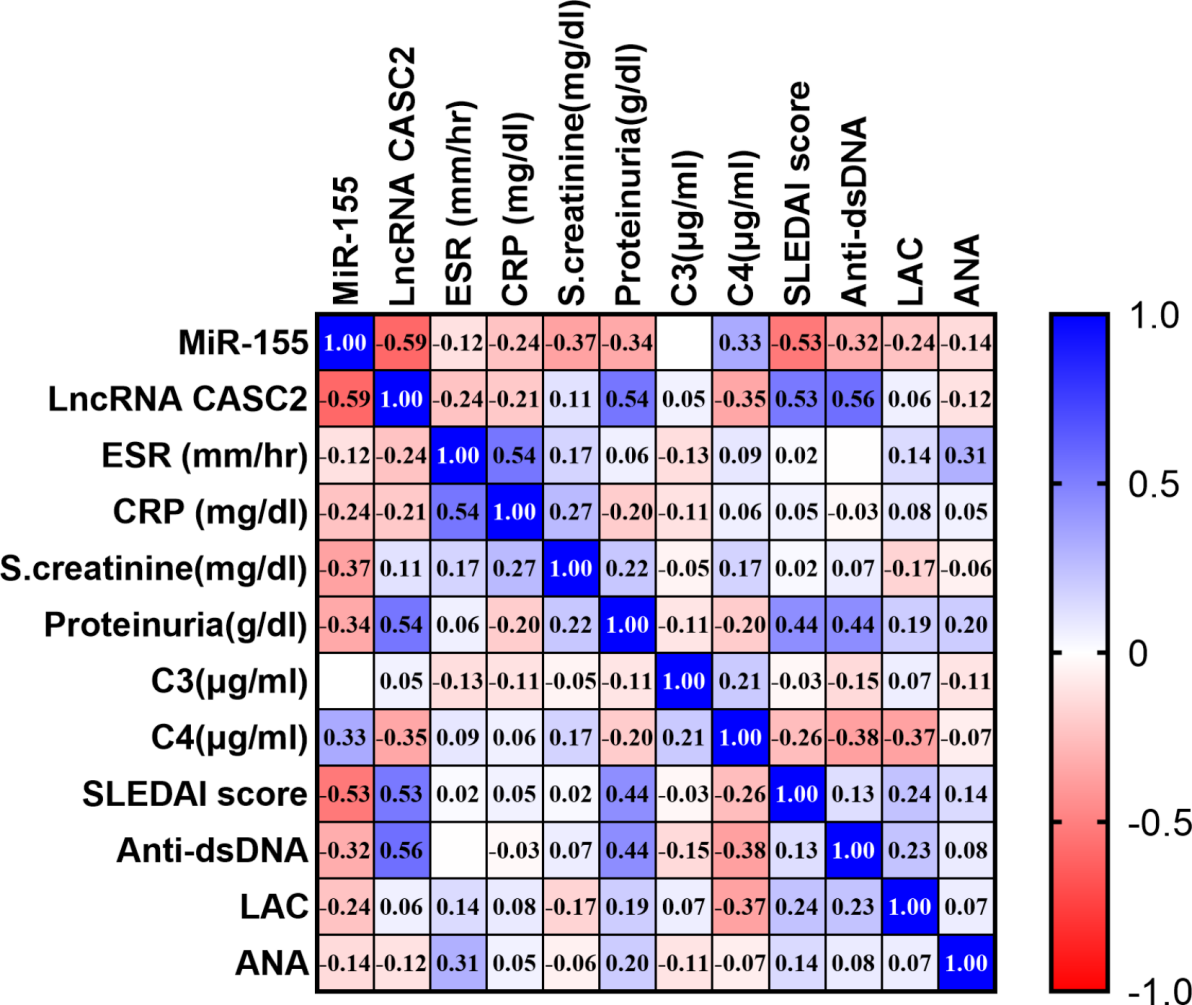
Spearman correlations among serum ncRNAs and laboratory variables in the LN group are illustrated on a correlation map. The map features a blue-red scale, with blue representing correlations near 1, red representing correlations near − 1, and white representing correlations around 0. Statistical significance at *P* < 0.05.

We assessed the correlation between serum lncRNA CASC2, miR-155 biomarkers, and other laboratory variables using the Spearman correlation method. In non-LN patients, lncRNA CASC2 exhibited a significant positive correlation with ESR (*r* = 0.43, *p* = 0.017), ANA (*r* = 0.49, *p* = 0.005), and SLEDAI (*r* = 0.48, *p* = 0.007). However, miR-155 had significant positive correlation to C3 (*r* = 0.43, *p* = 0.018), C4 (*r* = 0.5, *p* = 0.005), and significant inverse correlation to anti-dsDNA (*r* = − 0.64, *p* = 0.000) with no correlation to other parameters, as shown in (Fig. 5). In LN patients, lncRNA CASC2 had significant positive correlation to anti-dsDNA (*r* = 0.56, *p* = 0.001), proteinuria (*r* = 0.54, *p* = 0.002) and SLEDAI (*r* = 0.5, *p* = 0.003), while miR-155 showed a significant negative correlation with SCr (*r* = − 0.37, *p* = 0.04) and SLEDAI (*r* = − 0.53, *p* = 0.003). LncRNA CASC2 and miR-155 displayed a significant inverse correlation to each other (*r* = − 0.59, *p* = 0.001), as shown in (Fig. 6).

### Bioinformatics analysis

**Fig. 7:**
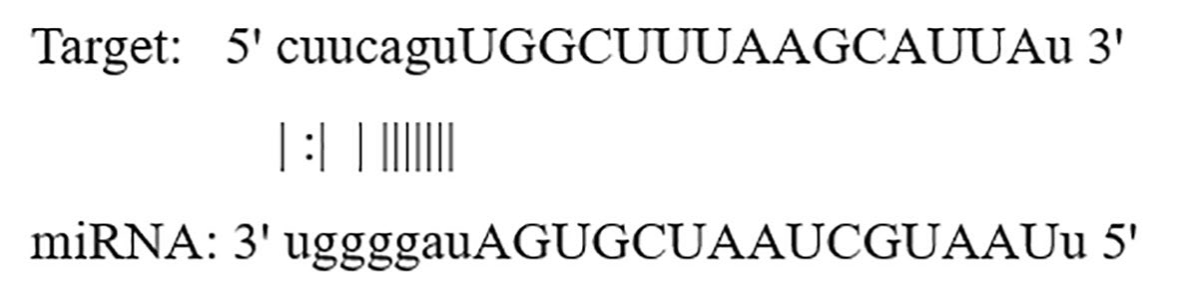
Prediction of lncRNA CASC2 and miR-155-5p interaction using the ENCORI database.

Through bioinformatics analysis, it was found that CASC2 shared binding sequences with miR-155 (Fig. 7), which is predicted by the ENCORI database.

Additionally, we utilized TargetScan to identify potential targets for miR-155-5p, revealing TGF-Beta Activated Kinase 1 Binding Protein 2 (TAB2) as a key biological target of miR-155-5p. The expected binding of miR-155-5p to the 3’ UTR of the TAB2 gene implies a functional regulatory potential. The binding site, in this case an 8mer, is known to have a strong binding affinity which results in a significant repression of gene expression. The context + + score of this interaction is -0.30 which lies in the 95th percentile. This means that of all the targeting miRNAs towards the TAB2 gene, miR-155-5p is the most abundant. Moreover, the dissociation constant value (KD) of -5.386 depicts a very high binding affinity thus it can be concluded that miR-155-5p has the right capability of repressing the expression of TAB2 and affecting other pathways involved in inflammation and immune response as presented in (Table 3).

**Table 3:** Predicted Interaction Metrics between Mir-155-5p and the TAB2 3’ UTR target site.

	Predicted consequential pairing of target region (top) and miRNA (bottom)	Site type	Context + + score	Context + + score percentile	*P*_CT_	Predicted relative K_D_
Position 818–825 of TAB2 3’ UTR hsa-miR-155-5p	5’ …GCCUUAAAAUAAAAAAGCAUUAA… ||||||| 3’ UGGGGAUAGUGCUAAUCGUAAUU	8mer	-0.30	95	0.49	-5.386

## Discussion

Delayed SLE diagnosis increases the risk of damage to vital organ systems, specifically renal involvement, which is termed LN. It is considered the primary predictor of both disease burden and mortality in SLE^43^. Consequently, recent studies have discussed a broad spectrum of emerging biomarkers for LN and their correlations to the disease’s pathophysiology and activity. Among them were growth factors, adhesion molecules, urine and serum cytokines, and non-coding RNAs like lncRNAs and miRNAs^44,45^.

The present study utilized at least four (SLICC) criteria to diagnose patients with SLE^37^, and renal involvement evaluated by SCr and proteinuria, which were significantly higher in LN patients than in non-LN patients (*p < 0.001)*. Significant increases in positive anti-dsDNA and LAC frequencies, and a significant reduction in serum C3 and C4 levels were also documented in SLE patients relative to the control group. However, these levels did not differ significantly between LN and non-LN patient groups.

This aligns with earlier research indicating that serum markers for SLE, like anti-dsDNA, LAC, and circulating complement factors (C3 and C4), are effective for diagnosis but have limitations in monitoring renal activity^46^. *Ahmed et al.*^47^ also proved that anti-dsDNA and LAC were positively detected in SLE patients compared to controls, and there was no statistically significant variation between patients with LN and those without. Similarly, *Chen et al.*^7^ found downregulation of circulating C3 and C4 in SLE patients, and this reduction did not vary significantly among LN and non-LN patients’ groups. In contrast, *Khoshmirsafa et al.*^48^. found a substantial difference in C3 and C4 levels between active LN and absent LN cases, but this distinction was not found in the current study.

Regarding clinical characteristics, our findings revealed no considerable variation among the LN and non-LN groups, except in the case of arthritis, which showed a significant variance (*p* < 0.05). This was consistent with the study that documented arthritis as the only clinical manifestation that significantly differentiates between LN and non-LN patients^47^, and against the study that reported that the LN group had a high significant prevalence of oral ulcers, photosensitivity, and discoid rash than the non-LN group (*p* < 0.001 for each)^49^.

Recent evidence suggests that the dysregulation of lncRNAs could be a fundamental factor in SLE development^50^. It has been demonstrated that in patients with SLE, plasma lncRNA levels were found to be increased for large intergenic noncoding RNA (linc0597) and decreased for lncRNA growth arrest-specific 5 (GAS5) and lnc-DC^10,51^. LincRNA-P21 also Participates in the pathogenesis of SLE by hindering the restoration of IL-2 mediated by miR-181a in LN^52^. Additionally, lncRNA metastasis-associated lung adenocarcinoma transcript 1 (MALAT1) shows predominant elevation in Monocytes and can directly influence the silent information regulator sirtuin 1 pathway, thereby enhancing the inflammatory response associated with SLE^53^. In our experiments, we reported for the first time that SLE patients’ serum concentration of the lncRNA CASC2 was significantly higher than those of healthy controls, with the highest values detected in LN patients versus the non-LN group (*P < 0.001)*. The increase in lncRNA CASC2 was also found to be associated with more advanced stages of LN. As a new indicator for SLE, CASC2 could be an important mediator in disease progression, particularly in discriminating between class II and IV. Supporting this, ROC analysis revealed lncRNA CASC2 as a promising diagnostic biomarker that could differentiate between non-LN and control groups with a 96.67% sensitivity and 100% specificity, also as a sensitive predictor to LN disease due to its ability to discriminate between LN and non-LN groups with an 86.67% sensitivity and 66.7% specificity. Additionally, the strong positive correlation between lncRNA CASC2 and SLEDAI in SLE patients indicates its role in detecting disease activity.

In accordance with an earlier investigation which noted a decrease in miR-155 in PBMCs taken from SLE patients, it was found that the amount of miR-155 expressed showed an opposite relationship with the levels of interferon alpha-17 (IFN-α17)^54^. *Wang et al.*^55^ also documented a decline in miR-155 levels in the serum and urine of SLE cases compared to controls, and this was consistent with the patient’s kidney function. Furthermore, miR-155-5p was found to be downregulated across all three murine lupus models^56^. The findings of this research reported a significant decline in MiR-155 expression in the SLE group as compared to the control group (*P* < 0.01) and in the LN patients’ group as compared to the non-LN group (*P* < 0.05). The downregulation of miR-155 was found to be associated with more advanced stages of LN, indicating its potential role in evaluating the disease progression, particularly in discriminating between class II and class (IV-V). This contradicts previous studies that reported increasing in miR-155 levels in SLE patients’ peripheral blood^57,58^. Furthermore, *Khoshmirsafa et al.*^48^ observed a significant elevation in miR-155 levels in the active LN cases compared to those with absent and inactive LN, and that there was a strong direct correlation between miR-155 levels and SLEDAI scores.

The differences observed between our findings and others might result from genetic or environmental factors, as well as different ethnicities among the populations that might alter miR-155 expression in SLE patients.

In our study, the diagnostic and predictive performance of miR-155 were assessed, reporting miR-155 as a powerful diagnostic biomarker with an 83.33% sensitivity and 100% specificity, but regarding its activity in predicting LN, lncRNA CASC2 showed superior predictive activity than it. This might be due to the lncRNA CASC2 regulatory role in modulating immune response, particularly through the production of proinflammatory cytokines like IL-6, IL17 and IL-18^24^, as well as, its role in regulating renal function as reported by previous literature that it could reflect the severity of acute kidney injury resulted from sepsis^16^. In addition to its potential power in prognosis and predicting the treatment response of inflammatory diseases^18,23^.

A strong correlation between miR-155 levels and the immunological specific markers including anti-dsDNA, C3 and C4 in the non-LN cases also was observed in our study, indicating its role in contributing to disease pathogenesis. In addition, miR-155 displayed an inverse correlation with both SCr and SLEDAI levels in LN patients, showing that it might have a vital role in mitigating LN disease severity. Supporting our findings, numerous research has documented a reverse relationship between miR-155 levels and SLEDAI score^59,60^. Additionally, the studies revealed an inverse relationship between the expression level of miR-155 and both anti-dsDNA antibodies and disease activity^61^, while showing a positive correlation with C3 and C4 levels in SLE patients^46^. Unlike the findings of some studies that indicated that there was a positive correlation between miRNA-155 and SLEDAI in SLE^46,48^.

Through bioinformatics analysis, it was found that CASC2 shared binding sequences with miR-155 as illustrated in **(**Fig. 7**)**. Dual luciferase reporter assays further confirmed the direct binding between miR-155 and lncRNA CASC2 in several studies^25,36,62,63^. As supported by these findings, we reveal lncRNA CASC2 as a ceRNA to sponge miR-155, and that both ncRNAs have the potential to be utilized as non-invasive diagnostic biomarkers. In addition to the powerful predictive activity of lncRNA CASC2 for LN disease.

**Fig. 8:**
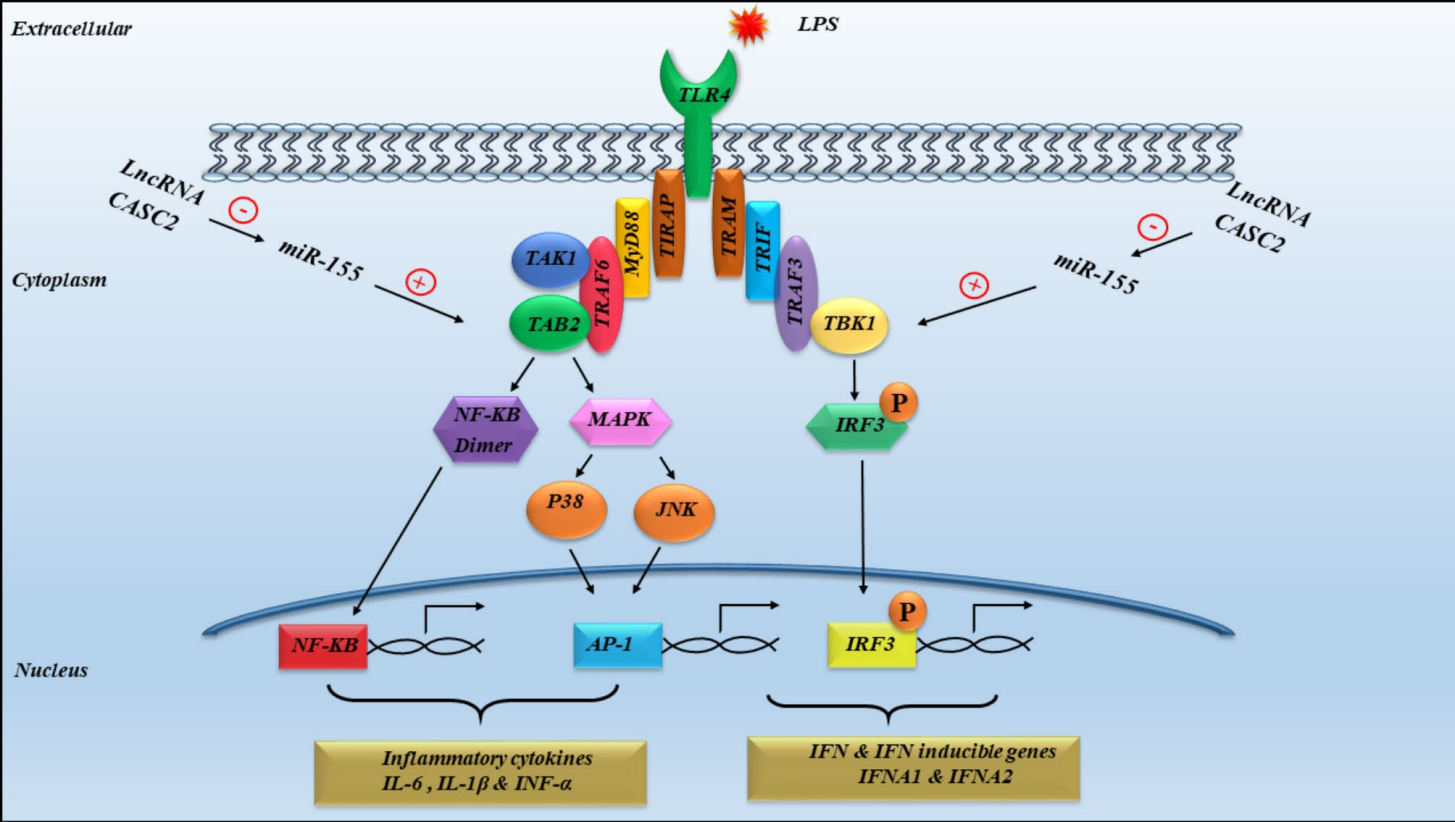
The lncRNA CASC2 downregulates miR-155, that indirectly increasing TAB2 and TBK 1 expression.

The explanation of these results is dependent on the impact of miR-155 downregulation on Toll-like receptor 4 (TLR-4) activation through two TLR-4 canonical signaling pathways dependent on the adaptor protein recruited as shown in (Fig. 8)^27,56^. The first is the myeloid differentiation primary response 88 (MyD88)-dependent pathway by targeting (MyD88/TAB2) cascades^27^. Several studies documented that TAB2 messenger RNA was inversely targeted by miR-155 in several inflammatory diseases such as SLE^27,64^, kidney disease^65^ and pulmonary disease^66^. TAB2 is established for its role in binding to and activating transforming growth factor-β-activated kinase-1 (TAK1), which then triggers various inflammatory pathways, such as NF-κB, p38 MAPK and c-Jun N-terminal kinase (JNK). This ultimately leads to the production of NF-κB and activator protein 1 (AP-1) transcription factors with inflammatory cytokines secretion like IL-6, IL-8, IL-1β, and TNF-α^67,68^.

The second pathway involves TIR domain-containing adaptor-inducing interferon-β (TRIF) activation, which is independent of MyD88. This activation triggers a cascade of transcription factors such as TRIF, TRIF-related adaptor molecule (TRAM), and TANK-binding kinase 1 (TBK1). Ultimately, this pathway leads to the production of the interferon regulatory factor 3 (IRF3), which then relocates to the nucleus to initiate the transcription of IFNA1 and IFNA2^56,69^. This is reinforced by previous studies that pointed to miR-155 as a negative regulator in type I IFN production in SLE^54,69^.

## Conclusion

This is the first research to evaluate the serum expression level of lncRNA CASC2 in patients with SLE (LN & non-LN). It showed a significant increase in lncRNA CASC2 expression in SLE cases specifically those with LN, in contrast to miR-155 expression, which was significantly reduced in LN patients relative to those without LN. Increased expression of lncRNA CASC2 and decreased expression of miR-155 were linked to more advanced stages of LN, highlighting their involvement in disease progression. Besides, we shed light on the direct relation between lncRNA CASC2 and miR-155 (act as a sponge) in the pathogenesis of SLE and represent them as non-invasive biomarkers that are used in the diagnosis and detection of disease severity of SLE, as well as their role in discriminating between LN and non-LN patients. However, lncRNA CASC2 is more sensitive than miR-155 in predicting LN disease.

Our investigation was limited in several ways: The modest sample size of our study might constrain its statistical power. Consequently, it is crucial to validate these findings with a larger and more diverse sample that includes more participants and various racial groups.

## Data Availability

All data generated or analysed during this study are included in this published article.
